# Supplementary material for: Causal Relationship between Aging and Anorexia Nervosa: A White-Matter-Microstructure-Mediated Mendelian Randomization Analysis
Source: Biomedicines. 2024 Aug 16;12(8):1874. doi: 10.3390/biomedicines12081874 (PMC11351342; doi:10.3390/biomedicines12081874)
Supplement: Supplementary file 1 [file biomedicines-12-01874-s001.zip › Supplementary Figure/Figure S8/Leaveoneout plot of MR analysis for TL to ubm-b-1641.pdf]

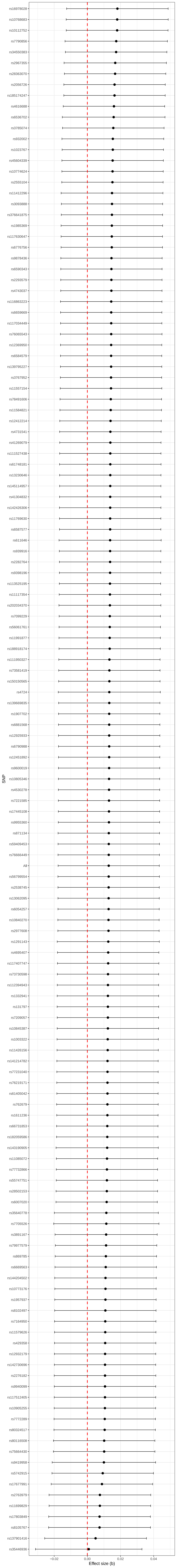

SNP
Effect size (b)
-0.02
0.00
0.02
0.04
rs16978028
rs10768683
rs10112752
rs7790856
rs34550383
rs2967355
rs28363070
rs2056726
rs185174247
rs4616688
rs6536702
rs3785074
rs932002
rs1023767
rs45604339
rs10774624
rs2555104
rs11412296
rs3093888
rs376641875
rs1985369
rs117630647
rs6776756
rs9878436
rs6590343
rs2293579
rs4743037
rs116863223
rs6659669
rs117034449
rs76065543
rs12369950
rs6584579
rs139795227
rs3767952
rs11557154
rs78491606
rs11584821
rs12412214
rs4731541
rs41269079
rs111527438
rs61748181
rs13230646
rs145114957
rs41304832
rs142426306
rs11769630
rs6587577
rs611646
rs939916
rs2282764
rs9398196
rs113525195
rs11117354
rs202034370
rs7099229
rs56061761
rs11991877
rs188918174
rs111950327
rs73581419
rs150150565
rs4724
rs139669835
rs1907702
rs6881568
rs12925933
rs6790988
rs12451892
rs9600019
rs10805346
rs4530278
rs7221585
rs17445108
rs9955360
rs871134
rs59409453
rs76666449
All
rs56799554
rs2538745
rs13062095
rs6054257
rs10840270
rs2977608
rs1291143
rs4695407
rs117407747
rs73730598
rs112394943
rs1332941
rs131797
rs7209057
rs10845387
rs1003322
rs11426156
rs141214782
rs77231040
rs76219171
rs61405042
rs762679
rs1611236
rs66731853
rs182059586
rs143190905
rs11085072
rs77732866
rs55747751
rs28502153
rs6007020
rs35640778
rs7705526
rs3891167
rs79977579
rs869785
rs6669563
rs144204502
rs10773176
rs1957937
rs8102497
rs7164950
rs11579626
rs429358
rs12932179
rs142730696
rs2276182
rs9940099
rs117512405
rs10905255
rs7772289
rs80324517
rs80116508
rs75664430
rs9419958
rs5742915
rs17677991
rs2763979
rs11699829
rs17803849
rs8105767
rs137901416
rs35446936
